# The Therapeutic Targets of Fingolimod (FTY720) Are Involved in Pathological Processes in the Frontal Cortex of Alzheimer's Disease Patients: A Network Pharmacology Study

**DOI:** 10.3389/fnagi.2021.609679

**Published:** 2021-02-02

**Authors:** Pengqi Yin, Yang Xue, Tingting Wang, Di Zhong, Guozhong Li

**Affiliations:** ^1^Department of Neurology, First Affiliated Hospital of Harbin Medical University, Harbin, China; ^2^Department of Pharmacology and Physiology, Georgetown University Medical Center, Washington, DC, United States; ^3^Interdisciplinary Program in Neuroscience, Georgetown University Medical Center, Washington, DC, United States

**Keywords:** Alzheimer's disease, fingolimod, S1PR1, network pharmacology, GABA synapses, frontal cortex, ALDH1L1

## Abstract

**Background:** The sphingosine-1-phosphate receptor (S1PR) modulator fingolimod (FTY720), which is commonly used as an immunomodulator in multiple sclerosis treatment, has recently been found to reduce pathological changes in the brain tissue of Alzheimer's disease (AD) animal models, but this has yet to be verified in human brain tissue. In this study, network pharmacology methods were applied to determine the potential pharmacological mechanisms of fingolimod in the frontal cortex of AD patients.

**Methods:** The pharmacological macromolecular targets of fingolimod and fingolimod phosphate were downloaded from SwissTarget and DrugBank. Systematic intersection analysis of the expression profiles of brain frontal cortex tissues (423 AD tissues and 266 control tissues) was performed to obtain AD-associated fingolimod targets (F-ADGs). Immune cell infiltration analysis and a primary mouse cortical culture RNA-seq drug screen database were used to identify immune-related F-ADGs and cortex-related F-ADGs. Then, the expression values of F-ADGs were correlated with the disease severity score (MMSE score) of AD patients to identify severity-related F-ADGs. We also analyzed miRNA expression microarray data in the frontal cortex of AD patients associated with disease severity to obtain severity-related F-ADG-miRNAs.

**Results:** A total of 188 F-ADGs were detected in the frontal cortices of AD patients and were enriched in biological processes such as synaptic signaling, inflammatory response, and response to oxygen-containing compounds. Eleven immune-related F-ADGs (like FPR1, BLNK.) and 17 cortex-related F-ADGs (like ALDH1L1, DUSP1.) were detected. Other F-ADGs, such as S1PR1 and GABBR2, although not classified into the above two categories, were still predicted by bioinformatics methods to play an important role in the development of AD. Two F-ADGs (GNAQ and MMP14) and 28 miRNAs (like miR- 323a-3p, miR-181a-5p.) were found to be associated with AD severity (MMSE 0-27 group). Fifteen F-ADGs (like ALDH1L1, FPR1, and IL6.) and 46 miRNAs (like miR-212-5p, miR-93-5p.) were found to be associated with mild or moderate dementia AD patients' severity (MMSE11-22 subgroup).

**Conclusions:** Fingolimod may affect the brain frontal cortex function of AD patients in many different ways, such as affecting immune cell infiltration, nerve cell, or glial cell function, and synaptic function. miRNAs may also be involved. ALDH1L1, FPR1, S1PR1, and GABBR2 may be core drug targets.

## Introduction

Fingolimod (FTY720), a sphingosine-1-phosphate receptor (S1PR) modulator, is the first oral drug approved by the Food and Drug Administration (FDA) for the treatment of relapsing-remitting multiple sclerosis (RRMS) (Brinkmann et al., 2010). Recently, fingolimod was reported to reduce the pathological changes in the brain tissue of Alzheimer's disease (AD) animal models (Angelopoulou and Piperi, 2019; Carreras et al., 2019; Jeśko et al., 2019), but this has yet to be verified in human brain tissues. The treatment effect of fingolimod in RRMS is commonly attributed to its ability to retain autoreactive lymphocytes in the lymph nodes and prevent them from entering the circulation and central nervous system (CNS) by downregulating the expression of S1PR1 on T lymphocytes (Hunter et al., 2016; Huwiler and Zangemeister-Wittke, 2017). However, as fingolimod is a lipophilic molecule, it can cross the blood-brain barrier (BBB) (Asle-Rousta et al., 2014; Hunter et al., 2016) and has been proven to exert direct effects in the CNS of RRMS patients and in animal models of degenerative diseases of the CNS, including inhibiting microglial activation, reducing astrocyte proliferation, reducing the loss of dendritic spines, and preventing excitotoxic neuronal death (Hunter et al., 2016). Since these pathophysiological processes have also been observed in AD patients, fingolimod is speculated to also have therapeutic effects on the pathological processes of AD patients.

To date, most of the studies that have explored the mechanism of action of fingolimod in AD animal models have focused on the effect of fingolimod on β-amyloid precursor protein (APP) metabolism and β-amyloid (Aβ) protein aggregation (Tonelli et al., 2010; Takasugi et al., 2011, 2013). However, due to the complexity of the pathologic processes of AD, current animal models cannot fully recapitulate AD, particularly late-onset disease. Therefore, in AD-related research, direct research in human brain tissue is necessary. However, there is currently no report directly demonstrating fingolimod function in the brain tissue of AD patients. Network pharmacology methods provide new approaches to more comprehensively elucidate the mechanisms underlying the pharmacological effects of drugs in specific diseases (Hopkins, 2008) and a possible technique to directly study human brain tissue. In this research, we used network pharmacology methods to determine the potential pharmacological mechanism underlying the treatment effect of fingolimod in AD patients and to identify potential targets of fingolimod in the brain tissues of AD patients.

## Materials and Methods

### Study Design and Rationale

**Figure 1:**
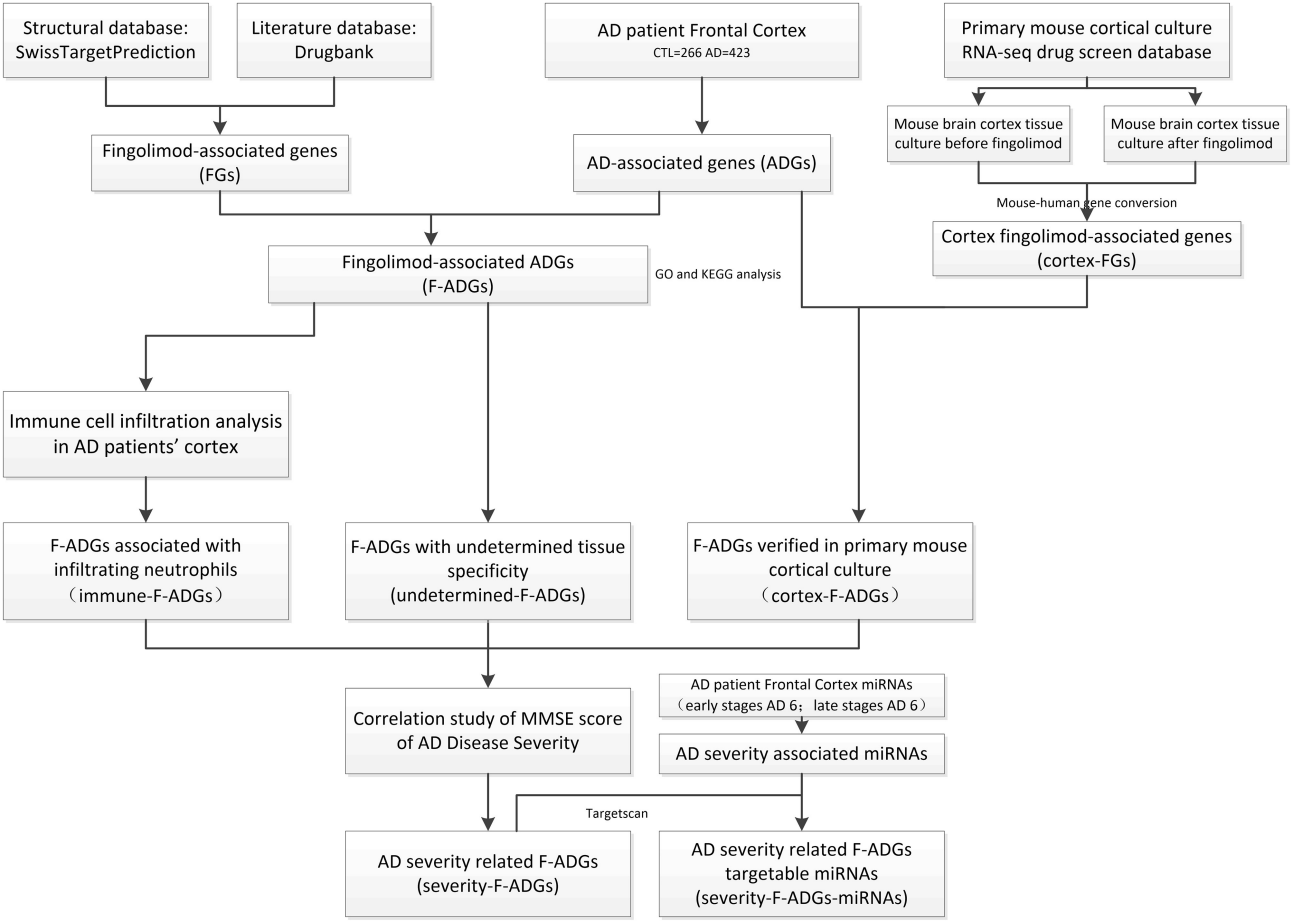
Flowchart of the analysis of the effect of fingolimod against AD. AD, Alzheimer's disease; CTL, control; FGs, fingolimod-associated genes; ADGs, AD-associated genes; F-ADGs, fingolimod-associated AD-associated genes.

In this study, the pharmacological macromolecular targets of fingolimod and fingolimod phosphate (i.e., fingolimod-associated genes, FGs) were downloaded from SwissTarget and DrugBank. Systematic intersection analysis of the expression profiles of brain frontal cortex tissues (423 AD tissues and 266 control tissues) was performed to obtain AD-associated genes (ADGs) and fingolimod AD-associated targets (F-ADGs). Immune cell infiltration analysis and a primary mouse cortical culture RNA-seq drug screen database were used to identify immune-related F-ADGs and cortex-related F-ADGs. Then, the expression values of F-ADGs were correlated with the disease severity score (MMSE score) of AD patients to identify severity-related F-ADGs. We also analyzed miRNA expression microarray data in the frontal cortex of AD patients associated with disease severity to obtain severity-related F-ADG-miRNAs. A flow chart of the experimental method is shown in Figure 1. In this study, we used the same method to analyze the data of the frontal cortex, temporal cortex and entorhinal cortex of AD patients. The main body of this article mainly used the frontal cortex data. The temporal cortex and entorhinal cortex data were shown in the Supplementary Materials.

### FGs

The chemical structures of fingolimod and its metabolic product fingolimod phosphate were downloaded from PubChem (https://pubchem.ncbi.nlm.nih.gov/). SwissTargetPrediction (Gfeller et al., 2013; Daina et al., 2019) (http://www.swisstargetprediction.ch/) was used to predict the pharmacological targets of fingolimod and fingolimod phosphate based on their chemical structures, and DrugBank (https://go.drugbank.com/) was used to collect experimentally confirmed fingolimod drug targets. In SwissTargetPrediction, pharmacological targets with a feasibility >0.1 were identified. UniProt (https://www.UniProt.org/) was used to standardize the naming of the drug targets. STRING (version 11.0) was used to retrieve protein-protein interaction (PPI) information with the retrieval condition “Species” set to “*Homo sapiens*,” the “confidence” set to “high (>0.7),” and the maximum number of interactions for the “1st shell” and “2nd shell” set to “no more than 20 interactors.” The target genes of fingolimod and fingolimod phosphate and the proteins that were predicted to interact with these target genes are collectively referred to as FGs.

### ADGs

Original microarray data associated with AD patients were retrieved from the Gene Expression Omnibus (https://www.ncbi.nlm.nih.gov/geo/) database by searching for the keyword “Alzheimer.” Exhaustive and non-redundant data for the frontal cortex were retrieved from eight datasets (GSE12685, GSE36980, GSE48350, GSE5281, GSE53890, GSE66333, GSE33000, and GSE118553) from 423 AD patients and 266 normal controls. All processed expression data were merged and then normalized with the “sva” package in R. Only genes (probes) for which <50% of the gene expression data were missing were retained. Then, the data from normal controls and AD patients were compared to identify ADGs. The “limma” package in R was used for differential expression analysis, and the Benjamini-Hochberg's method was used to correct for multiple comparisons. A |fold change| >1.2 and *p* < 0.05 were considered to indicate significantly differentially expressed genes.

### F-ADGs, PPI Analysis, and Gene Function Analysis

The FGs and ADGs were compared to identify AD-associated fingolimod targets (F-ADGs). STRING (version 11.0) was used for PPI analysis. After repeats were eliminated, a “confidence score” >0.7 was set to design the PPI network. Gene Ontology (GO) and Kyoto Encyclopedia of Genes and Genomes (KEGG) pathway analyses of F-ADGs were performed using WebGestalt (http://www.webgestalt.org/) (Liao et al., 2019). The following parameters were used in the enrichment analysis: minimum number of IDs in the category: 5; maximum number of IDs in the category: 2000; FDR method: BH; significance level: Top 20 and *p* < 0.01.

### Analysis of Immune Cell Infiltration in the Frontal Cortices of AD Patients

CIBERSORTx (https://cibersortx.stanford.edu/) (Newman et al., 2019) was used to estimate the percentages of various cell populations in the cortex of each AD patient. A signature matrix of each cell type was generated with the default settings. Bulk RNA-seq data were deconvoluted using the signature matrix with S-batch correction to remove variances between different sequencing platforms. Two-tailed unpaired Student's *t*-test was used to analyse differences in the abundances of various cell types between AD patients and normal controls. The *p*-values were corrected for multiple testing using the Benjamini-Hochberg method. *P* < 0.05 was considered to indicate statistical significance.

### Cortex-Related FGs and Cortex-Related F-ADGs

To better identify the direct effects of fingolimod on the nervous system and eliminate interference by immune infiltration, we used the “Primary mouse cortical culture RNA-seq drug screen database” (http://bigbear.med.uottawa.ca:1000/) (Hadwen et al., 2018). We compared the gene expression profiles of mouse brain cortex tissue cultures before and after treatment with fingolimod and obtained mouse cortex-related fingolimod-associated genes (cortex-related FGs). Then, we used Mouse Genome Informatics (http://www.informatics.jax.org/) to transform the mouse gene sets into the corresponding human gene sets and obtained the cortex-related fingolimod-associated genes (cortex-related FGs). Then, we used the intersection of cortex-related FGs and ADGs to obtain cortex-related F-ADGs.

### Analysis of the Undetermined F-ADGs

The F-ADGs that were not classified as immune-related F-ADGs or cortex-related F-ADGs were defined as undetermined F-ADGs. The Minimal Common Oncology Data Elements (MCODE) method was used for network analysis to select core molecular modules from the PPI network. The screening criterion for the core modules was twice the average degree of the “MCODE score.” The degree cut-off was set to 2, the node score cut-off was set to 0.2, the K-core was set to 2 and the maximum depth was set to 100. Undetermined F-ADGs with MCODE scores > 16 were considered key genes and were subjected to GO functional analysis.

### Correlation Analysis of F-ADG Expression and the Clinical Severity of AD

The frontal cortex expression profiles of 14 AD patients were correlated with their Mini-Mental State Exam (MMSE) scores, which ranged from 0 to 27. At the same time, we conducted a stratified analysis of subgroups of patients with mild and moderate dementia (MMSE score 10–23). In this study, 9 patients with MMSE scores from 11–22 were collected in this subgroup. Pearson's correlation analysis was used to assess the correlation between the expression values of the F-ADGs and the MMSE scores of the AD patients. *R* > 0.7 indicated a very strong linear correlation, 0.5 < *R* ≤ 0.7 indicated a significant linear correlation, 0.3 < *R* ≤ 0.5 indicated a low linear correlation and *R* ≤ 0.3 indicated no linear correlation. *P* < 0.05 was considered statistically significant.

### Analysis of the miRNAs Targeted by AD Severity-Related F-ADGs

First, we downloaded the GSE48552 dataset from the GEO database, which contains miRNA expression data of the frontal cortices of 6 early-stage AD patients and 6 late-stage AD patients. Then, we compared the frontal cortex miRNA expression data of early- and late-stage AD patients to obtain miRNAs related to the severity of AD. TargetScan (http://www.targetscan.org/vert_71/) (Lewis et al., 2005) is widely used to predict possible targets of miRNAs by pairing conserved 8-mer, 7-mer, and 6-mer sites within the seed region of miRNAs. In this study, the severity-related F-ADGs were applied to TargetScan to predict possible targeted miRNAs. Then, we intersected these possible targeted miRNAs with the AD severity-associated miRNAs to obtain the severity-related F-ADG-miRNAs.

## Results

### FGs

A total of 36 pharmacological targets of fingolimod or fingolimod phosphate (4 confirmed and 32 predicted based on chemical structures) from the TargetScan database and 11 targets from the DrugBank database were identified. Using the STRING database, 1,054 proteins that interact with these targets were identified and designated FGs (Supplementary Material 1).

### ADGs

A total of 2,149 genes that were differentially expressed in the frontal cortices of AD patients compared to healthy controls were identified as ADGs; 1,046 of these genes were upregulated and 1,103 were downregulated in AD patients compared to normal controls (Supplementary Material 2). At the same time, we also analyzed the data of the temporal and entorhinal cortexes of AD patients in the same way (Supplementary Material 2, Supplementary Figure 1).

### F-ADGs

**Figure 2:**
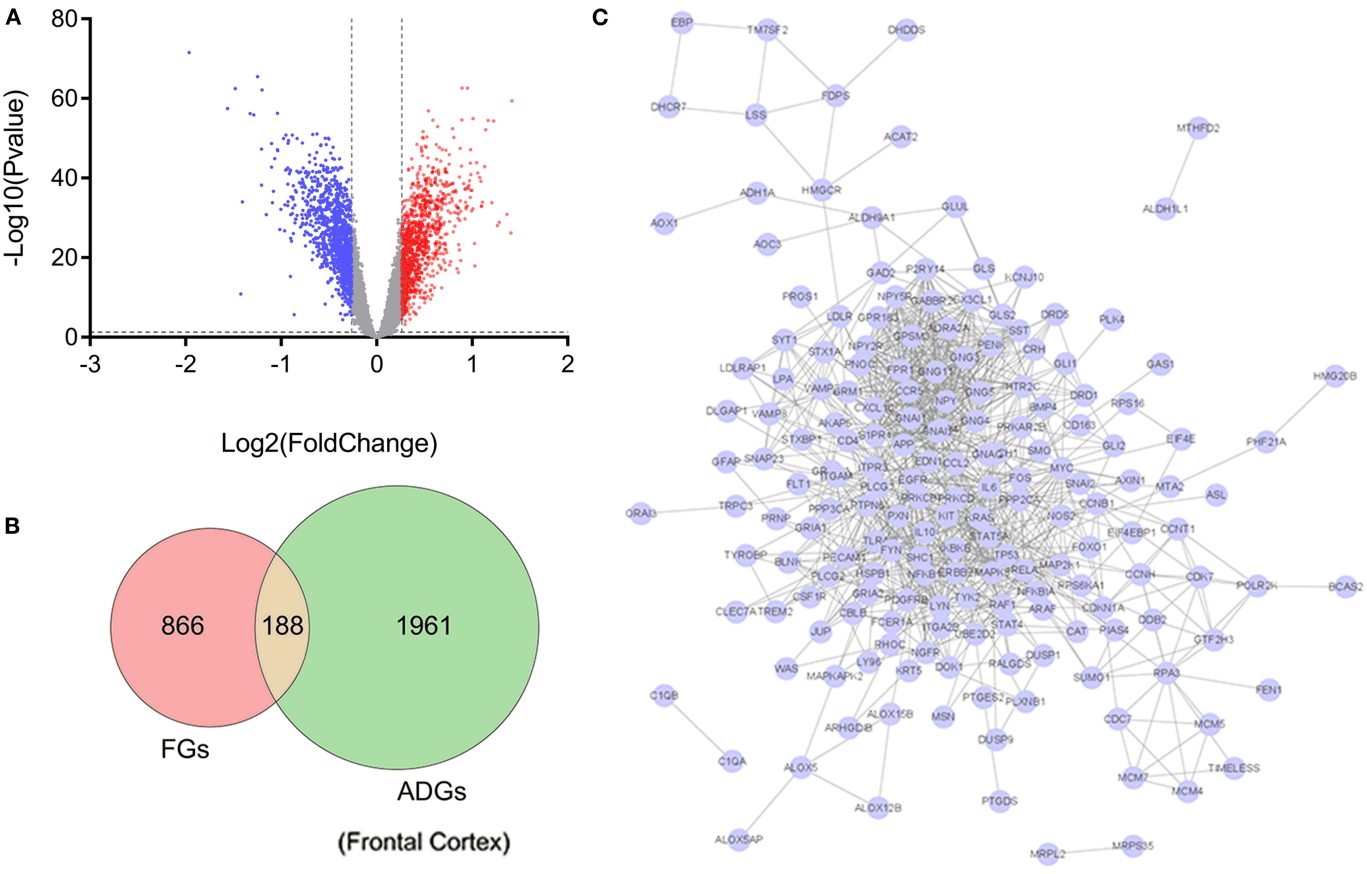
Pharmacological targets of fingolimod and FGs. **(A)** Volcano map showing the differentially expressed genes in the frontal cortices of AD patients compared to normal controls. Significantly upregulated genes are shown in red, significantly downregulated genes are shown in blue, and unchanged genes are shown in gray. **(B)** Venn diagram showing FGs, ADGs, and F-ADGs. **(C)** PPI networks of F-ADGs. Each node represents an F-ADG.

Of the 2,156 ADGs identified in the frontal cortex, 164 genes (100 upregulated and 64 downregulated) were FGs, suggesting that they are targets of fingolimod and were designated F-ADGs. A total of 575 interacting edges were formed consisting of 110 F-ADGs in the PPI network (Figure 2, Supplementary Material 3).

### Gene Function and KEGG Pathway Enrichment Analysis of F-ADGs

**Figure 3:**
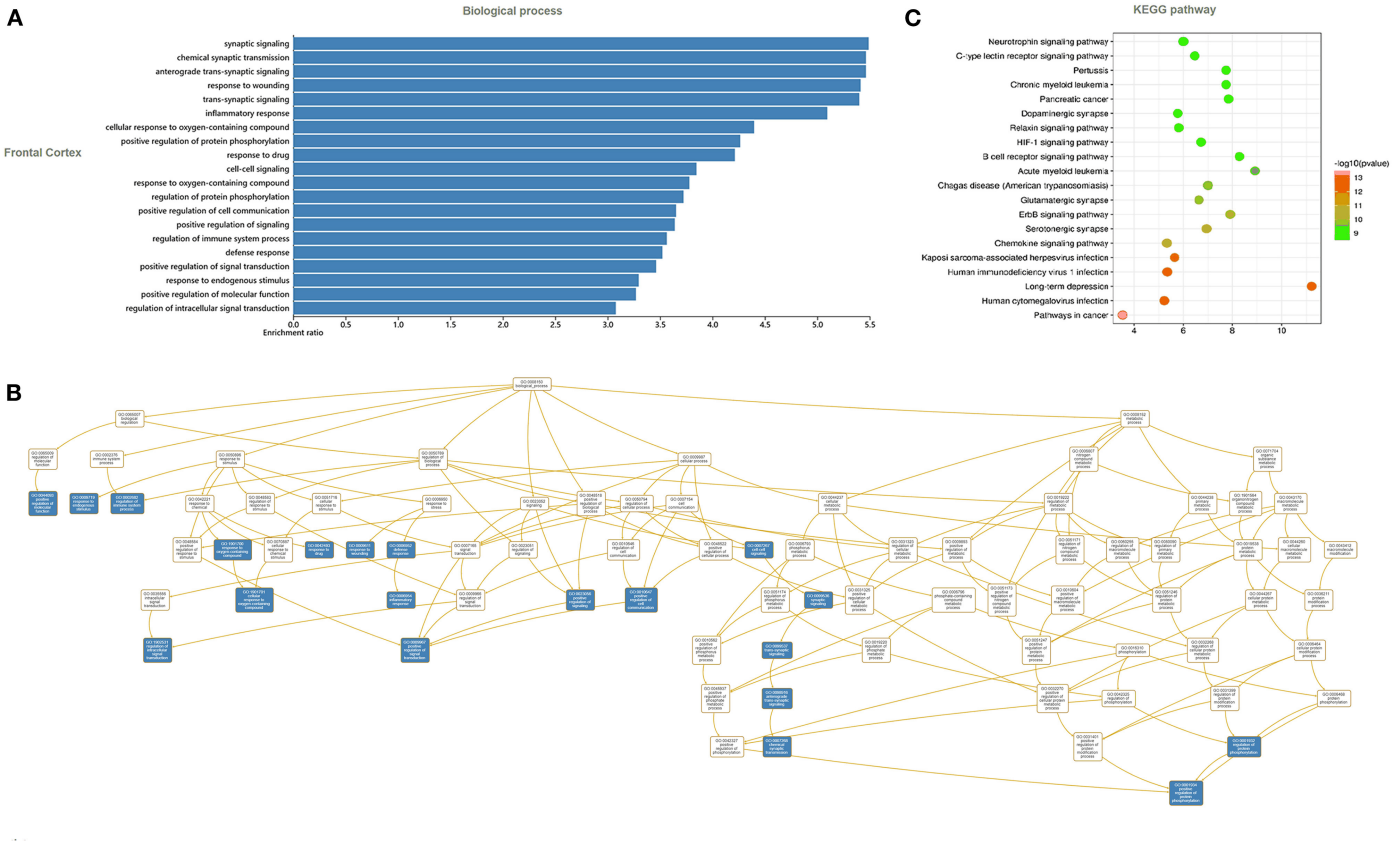
GO and KEGG analyses of F-ADGs. **(A)** Histogram showing GO enrichment analysis of F-ADGs. **(B)** Directed acyclic graph showing GO enrichment analysis of F-ADGs. **(C)** Bubble chart showing KEGG pathway enrichment analysis of F-ADGs.

In the frontal cortex, the 188 identified F-ADGs were enriched in biological processes such as synaptic signaling, inflammatory response, and response to oxygen-containing compounds, and KEGG pathways such as neurotrophin signaling pathways, dopaminergic synapses, and glutamatergic synapses (Figure 3, Supplementary Material 4).

### Immune-Related F-ADGs

**Figure 4:**
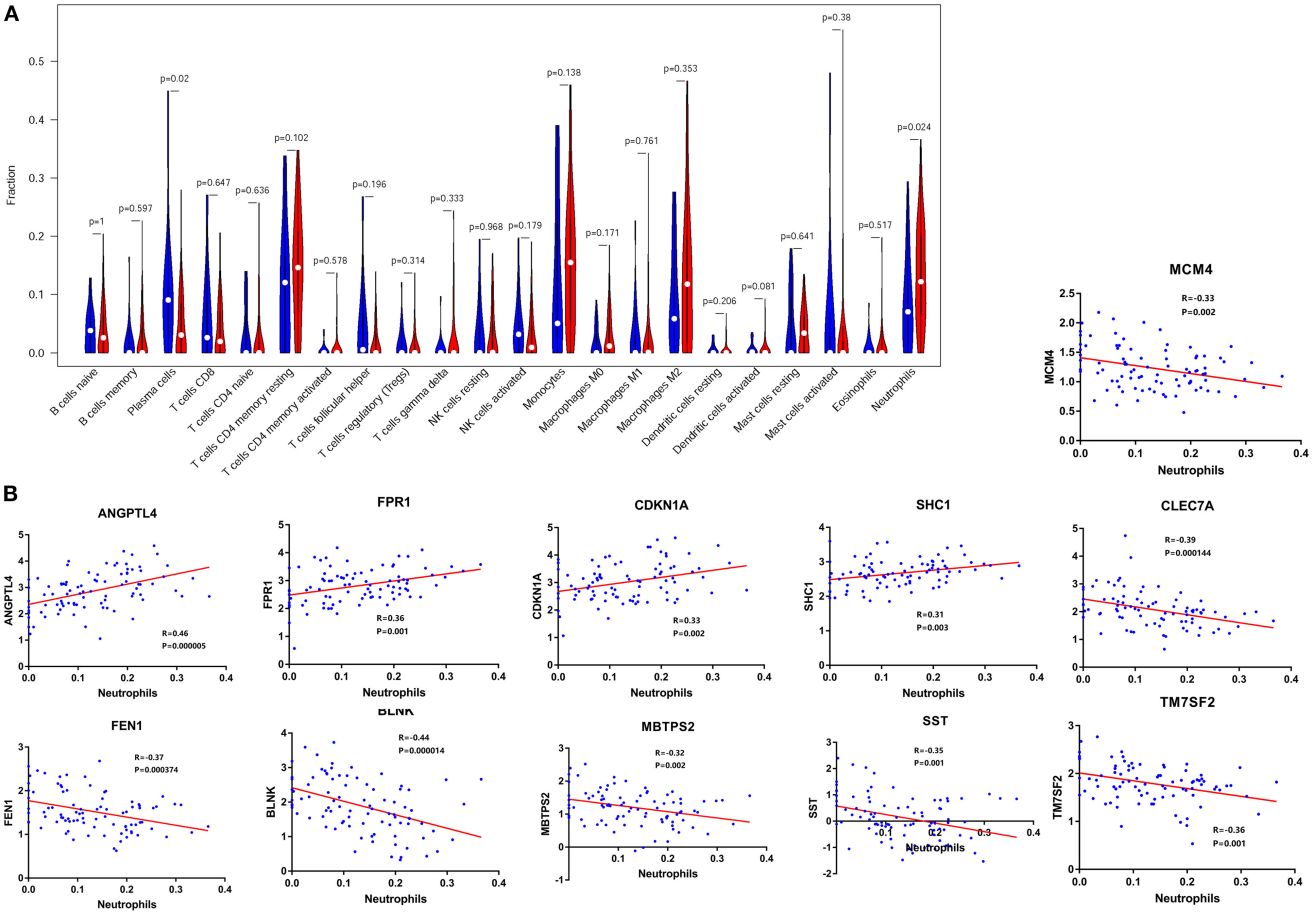
Neutrophil-related F-ADGs. **(A)** Violin plot of frontal cortex immune cell infiltration in AD patients. **(B)** Correlation analysis of the expression levels of neutrophil-related F-ADGs and neutrophil signals. The blue dots indicate the expression levels of the genes in each sample.

Immune infiltration analysis showed that compared with normal controls, AD patients had higher neutrophil infiltration (*p* < 0.05) and lower plasma cell infiltration (*p* < 0.05) in the frontal lobe brain tissue. By comparing the signal intensity of infiltrating cells with the expression signal intensity of F-ADGs, we found that no F-ADGs showed a correlation with plasma cells, while 11 F-ADGs showed a correlation with neutrophils. Among the 11 F-ADGs related to neutrophils, ANGPTL4, FPR1, CDKN1A, and SHC1 were positively correlated with neutrophil infiltration, while MCM4, CLEC7A, FEN1, BLNK, MBTPS2, SST, and TM7SF2 were negatively correlated with neutrophil infiltration. We believe that the four genes that are positively related to the signal intensity associated with neutrophil infiltration may have a more direct correlation with neutrophil infiltration and are more likely to be targets of fingolimod that regulate the infiltration of neutrophils in the frontal lobe of AD patients (Figure 4, Supplementary Material 5).

### Cortex-Related F-ADGs

**Figure 5:**
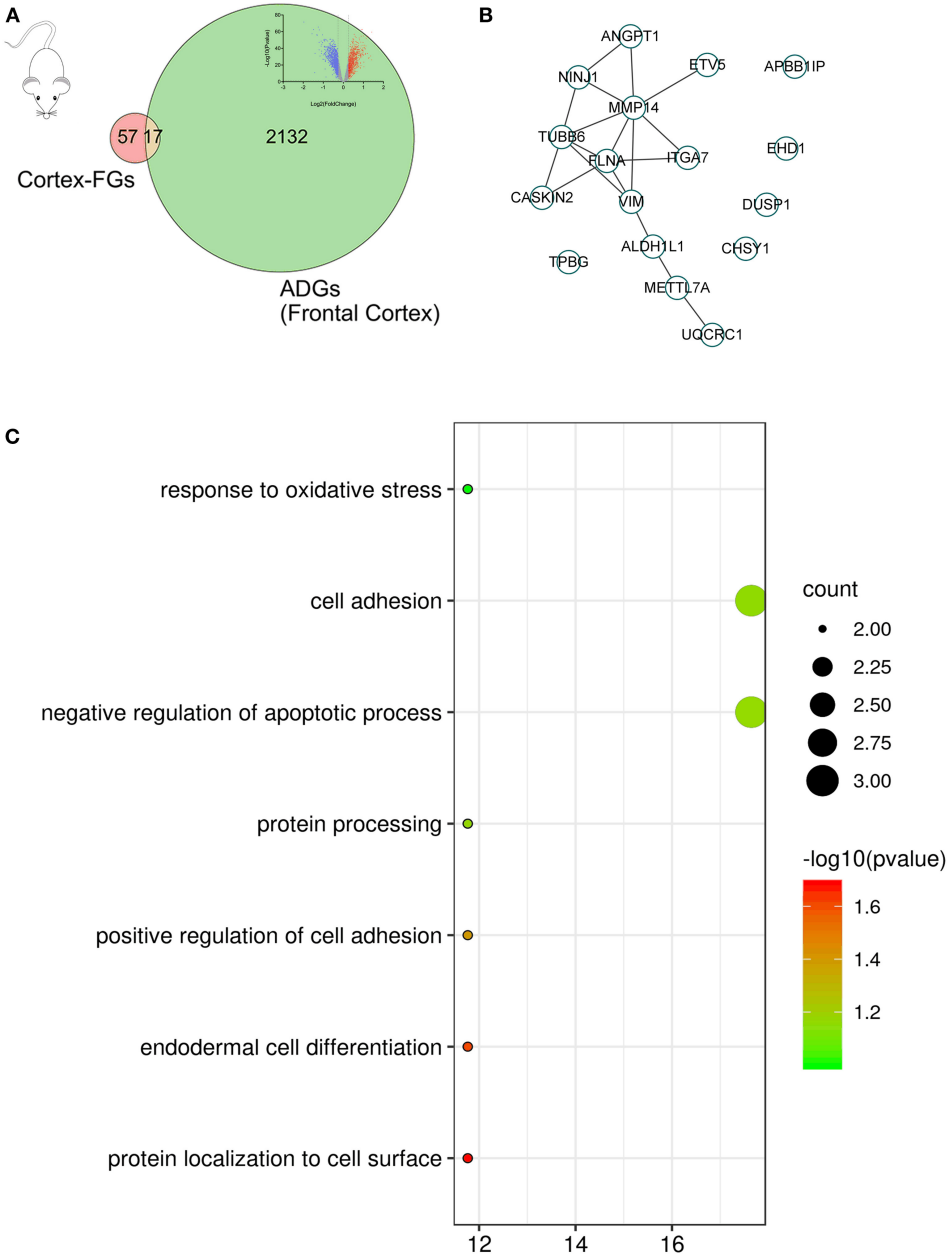
Cortex-related F-ADGs. **(A)** Venn diagram showing cortex-related FGs, ADGs, and cortex-related F-ADGs. **(B)** PPI network of cortex-related F-ADGs. **(C)** GO enrichment analysis of cortex-related F-ADGs.

In the primary mouse cortical culture RNA-seq drug screen database, we compared the mouse brain cortex expression data before fingolimod and after fingolimod treatment, conducted mouse-human gene name conversion, and obtained 74 cortex-related FGs. Then, we compared the cortex-related FGs with ADGs and obtained 17 cortex-related F-ADGs. The PPI analysis of cortex-related F-ADGs showed that VIM, MMP14, and FLNA were key genes in the network, suggesting that these genes may be targets of fingolimod that act directly on the nerve tissue of AD patients. GO analysis showed that the cortex-related F-ADGs were enriched in functions such as response to oxidative stress, cell adhesion, and negative regulation of apoptotic process (Figure 5, Supplementary Material 6).

### Undetermined F-ADGs

**Figure 6:**
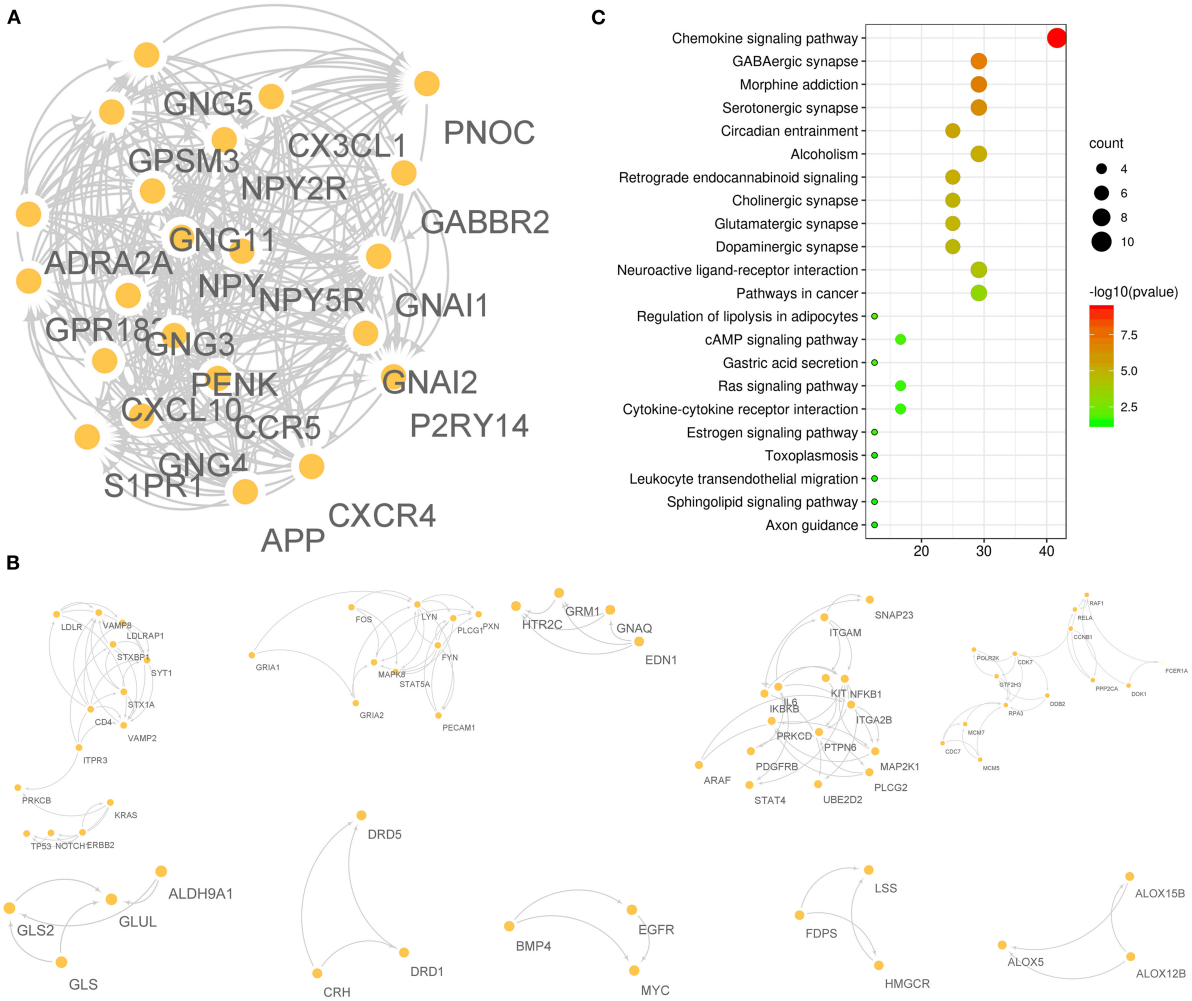
Undetermined F-ADGs. **(A)** PPI network of the 1st gene group of undetermined F-ADGs. **(B)** PPI network of the 2nd−11th gene groups of undetermined F-ADGs. **(C)** KEGG enrichment analysis of undetermined F-ADGs.

A total of 175 F-ADGs were classified as undetermined F-ADGs. The MCODE method divided undetermined F-ADGs into 11 parts, and the genes in the first part showed the strongest functional correlation. It is worth noting that S1PR1, the classic target of fingolimod, was also included in the first part, which suggested that these functionally related genes may be regulated by fingolimod at the same time. Functional enrichment analysis showed that the undetermined F-ADGs in the first part were enriched in KEGG pathways such as the chemokine signaling pathway, GABAergic synapses, and morphine addiction (Figure 6, Supplementary Material 7).

### Severity-Related F-ADGs

The correlation between the MMSE score of 14 AD patients, which ranged from 0 to 27, was collected. Two F-ADGs (GNAQ and MMP14) were found to be correlated with MMSE scores and were designated severity-related F-ADGs.

**Figure 7:**
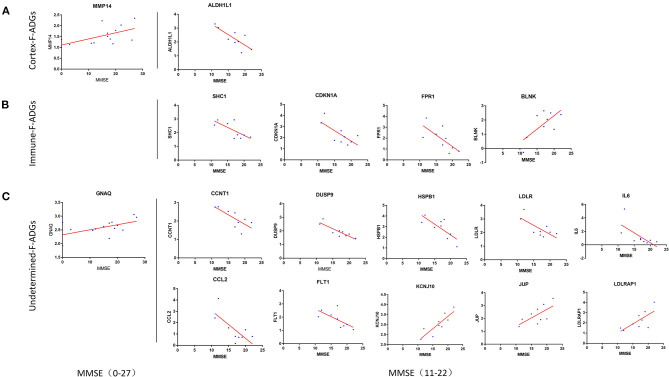
Severity-related F-ADGs **(A)** Cortex-related F-ADGs, **(B)** immune-related F-ADGs, and **(C)** undetermined F-ADGs. Correlation analysis of the expression levels of F-ADGs and MMSE scores as a measure of AD clinical severity. Each group includes F-ADGs from MMSE (0–27) group and F-ADGs from MMSE (11–22) subgroup. MMSE scores are plotted on the x-axis. Gene expression values are plotted on the y-axis. The blue dots indicate the expression levels of the core genes in each sample.

At the same time, we conducted a stratified analysis of subgroups of patients with mild and moderate dementia (9 patients with MMSE scores from 11 to 22). The correlation between the MMSE score of 9 AD patients, which ranged from 11 to 22, was collected. Fifteen F-ADGs were found to be strongly correlated with MMSE scores and were designated severity-related F-ADGs. Among the cortex-related F-ADGs, ALDH1L1 was positively correlated with the severity of AD (negatively correlated with the MMSE score). Among the immune-related F-ADGs, SHC1, CDKN1A, and FPR1 were positively correlated with the severity of AD, while BLNK was negatively correlated with the severity of AD. Among the undetermined F-ADGs, CCNT1, DUSP9, HSPB1, IL6, LDLR, CCL2, and FLT1 were positively correlated with the severity of AD, while KCNJ10, JUP and LDLRAP1 were negatively correlated with the severity of AD (Figure 7, Supplementary Material 8).

### Severity-Related F-ADG-miRNAs

**Figure 8:**
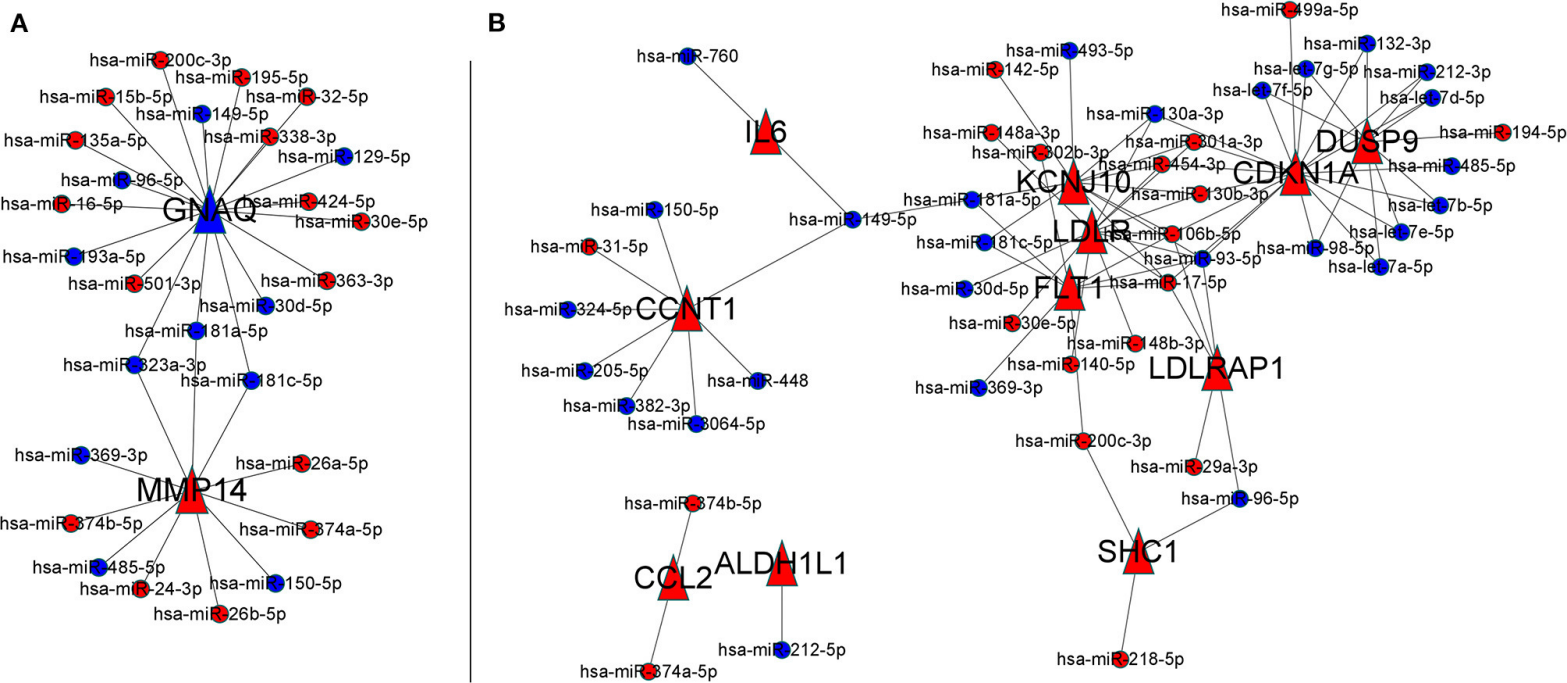
Severity-related F-ADG-miRNAs **(A)** AD patients with MMSE score 0–27 group, **(B)** mild or moderate dementia AD patients subgroup (MMSE score 11–22). The red dots indicate miRNAs that are upregulated in late AD; the blue dots indicate miRNAs that are downregulated in late AD; and the red triangle indicates the mRNA that is upregulated with the worsening of AD.

A total of 270 miRNAs were found to be differentially expressed in the frontal cortex in the early and late stages of AD. Among them, 169 had lower expression in the early stage of AD, and 101 had higher expression. These miRNAs were recorded as AD severity-associated miRNAs. In MMSE score 0–27 group, the 2 severity-related F-ADGs (GNAQ and MMP14) were found to be targeted by 28 miRNAs (like miR- 323a-3p and miR-181a-5p.); In mild and moderate dementia AD subgroup (MMSE scores from 11 to 22), among the 15 severity-related F-ADGs obtained above, 11 were found to be targeted by 46 AD severity-associated miRNAs. These AD severity-associated miRNAs were recorded as severity-related F-ADG-miRNAs (Figure 8, Supplementary Material 9).

## Discussion

**Figure 9:**
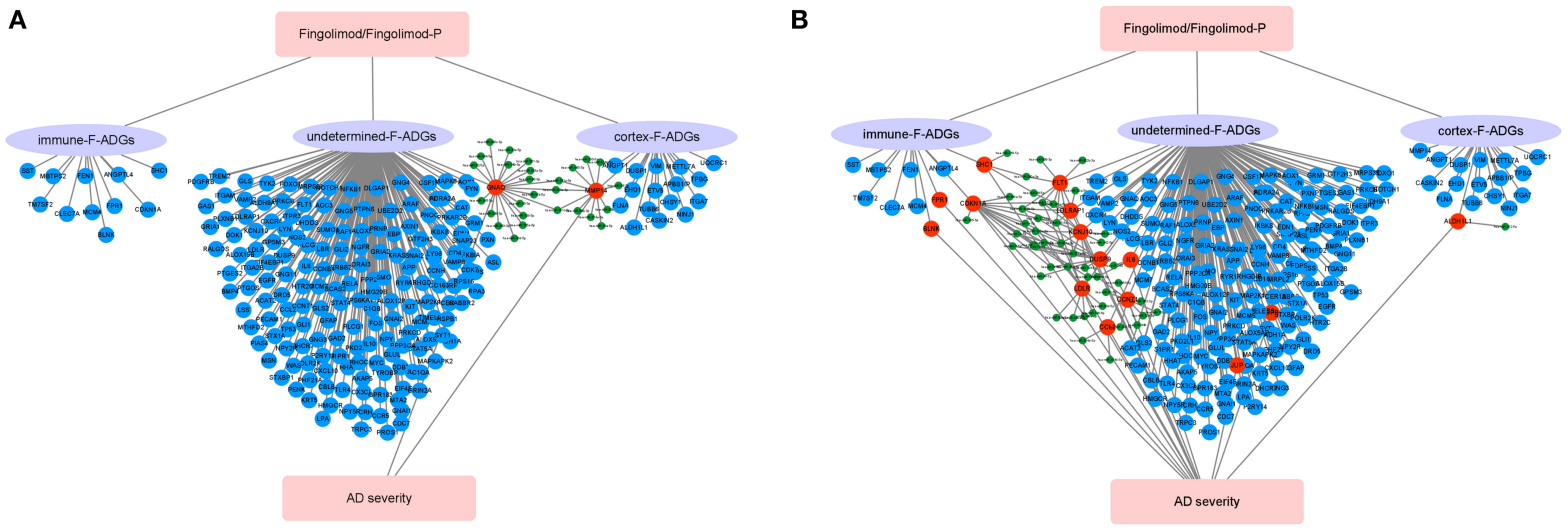
Schematic diagram of the synaptic action of fingolimod in AD patients. **(A)** AD patients with MMSE score 0-27 group, **(B)** mild or moderate dementia AD patients subgroup (MMSE score 11-22). The red circles represent F-ADGs that are related to the severity of AD; the blue circles represent F-ADGs that are not related to the severity of AD; and the green circles represent miRNAs.

AD is the most common cause of dementia in the world, and there are no effective therapies that can halt disease progression (Rossor, 1993; Brookmeyer et al., 2018; Weller and Budson, 2018). The S1PR modulator fingolimod (FTY720), which is an immunomodulator used in the treatment of multiple sclerosis, was recently reported to reduce the pathological changes in the brain tissue of AD animal models, but this has yet to be verified in human brain tissue. In this study, we used network pharmacology to identify possible fingolimod drug targets in the frontal cortex of AD patients. We found that fingolimod may affect the pathological processes in the CNS of AD patients by affecting the number and function of neurons and astrocytes, the infiltration of neutrophils in the frontal cortices, cell apoptosis, GABA synaptic function, and miRNA interactions, among others (Figure 9).

ALDH1L1 is one of the key drug targets discovered in this study. ALDH1L1 was predicted to be targeted by fingolimod in both the human drug target database and mouse cortex drug target database. Since the correlation between this gene and fingolimod has been reported in studies of these two different species, we believe that the discovery has a high degree of credibility. Since ALDH1L1 is commonly viewed as a pan-astrocytic constitutive marker (Yoon et al., 2017), it is possible that fingolimod may increase the number of astrocytes in the CNS. However, it is worth noting that the increase in the number of astrocytes is a double-edged sword for AD patients. On the one hand, maintenance of the number of healthy astrocytes is necessary for stable brain function, including regulation of synaptic activity, neuronal metabolism, and regional blood supply. On the other hand, overproliferation of astrocytes may aggravate a series of pathological damages in AD patients, including glutamate excitotoxicity, impaired synaptic plasticity, reduced carbon delivery to neurons for oxidative phosphorylation, and dysregulated linkages between neuronal energy demand and regional blood supply. Therefore, we cannot conclude whether this potential function of fingolimod in mediating astrocyte proliferation mentioned above is beneficial or harmful to AD patients (Habib et al., 2020). Similarly, as the results of the severity-related F-ADGs of this study also suggested that the expression level of ALDH1L1 was positively correlated with the severity of AD, it is unclear whether this would be a beneficial compensation mechanism or a cause of aggravation. In addition, it is worth noting that in this study, we found that miR-212-5p, which has a mutual targeting relationship with ALDH1L1, has a lower content in brain tissue in late AD than in early AD, which is consistent with the trend that ALDH1L1 expression increases with the severity of the disease. However, because a lower miR-212 level has been proven to protect elderly patients from dementia (Hadar et al., 2018), we believe that the elevated ALDH1L1 in AD patients may reduce the level of miR-212 in the brain tissue by acting as a miRNA sponge to play a neuroprotective function, and fingolimod may increase this beneficial function by increasing ALDH1L1 levels in brain tissue.

Another key target proposed in this study is DUSP1. This gene was also predicted to be regulated by fingolimod in both the human database and the mouse data cortical culture database. Data from the mouse database showed that the expression of DUSP1 in the cerebral cortex of mice increased after fingolimod treatment. At the same time, the expression of DUSP1 in the temporal cortices and entorhinal cortices of AD patients was also found to be higher than that of normal controls (Supplementary Figure 2). In the human cortex, DUSP1 protein expression correlates with tau phosphorylation (Arango-Lievano et al., 2016), synaptic defects and cognitive decline in subjects diagnosed with AD. Therefore, if fingolimod can truly increase the expression level of DUSP1 in the brain tissue of AD patients, it is reasonable to believe that fingolimod can affect the pathogenesis of AD by regulating tau phosphorylation.

Among immune-related F-ADGs were 11 gene targets related to neutrophil infiltration. Among them, the four genes ANGPTL4, FPR1, CDKN1A, and SHC1 were positively correlated with neutrophil signaling, and we believe that these genes are more likely to be directly related to neutrophil infiltration. Angiopoietin-like 4 (ANGPTL4) is a hypoxia-induced factor that is upregulated and secreted by human cortical astrocytes under hypoxic conditions (Chakraborty et al., 2018). Therefore, the increased expression of ANGPTL4 in AD patients is consistent with the increase in the expression of the abovementioned astrocyte-related genes in the brain tissue of AD patients compared to normal controls. ANGPTL4 can increase vascular permeability (Gomez Perdiguero et al., 2016), so fingolimod may regulate inflammatory cell infiltration in the CNS by targeting ANGPTL4. Formyl peptide receptor (FPR) is a G protein-coupled chemokine receptor that is mainly expressed in phagocytic leukocytes (Krepel and Wang, 2019). In related studies on colorectal tumors (Li et al., 2017), FPR1 mRNA expression has been confirmed to be related to tumor serous membrane infiltration, and FPR1 protein is expressed in colorectal epithelium and tumor-infiltrating neutrophils/macrophages (Chen et al., 2017). In this study, it was found that the FPR1 signal in AD brain tissue increased, which was related to neutrophil infiltration and was positively correlated with the severity of AD. Therefore, if fingolimod can reduce the expression of FPR1 as predicted in this study, it may improve the occurrence and development of AD by reducing the adverse effects of immune infiltration.

Dysfunction of the GABAergic system may cause cognitive impairment in humans (Solas et al., 2015). In severe AD patients, GABA levels have been shown to be significantly reduced, which may be the cause of AD behavior and psychological symptoms (Calvo-Flores Guzmán et al., 2018). Increasing evidence supports the remodeling of GABAergic synapses in the AD brain, which may begin in the early stages of disease pathogenesis last through the entire course of AD (Mandal et al., 2017). Therefore, GABAergic synapses may become effective molecular targets for AD drug development and pharmacological treatment. In this study, the functional enrichment of F-ADGs resulted in the enrichment of GABAergic synapses, which suggests that fingolimod may exert pharmacological effects on GABA synapses that undergo pathological changes during the pathogenesis of AD. It is worth noting that in the undetermined F-ADG group analysis, S1PR1, the currently recognized main target of fingolimod, was found to be closely related to GABA synaptic function. In addition, the expression of S1PR1 was found to be increased in the frontal cortices, temporal cortices, and entorhinal cortices of AD patients, which suggests that S1PR1 is widely involved in the pathological changes of the AD cerebral cortex (Supplementary Material 2, 3, Supplementary Figure 2). Therefore, in the CNS, fingolimod phosphate may act by directly targeting S1PR1 on the surface of the CNS and may act by influencing GABAergic synaptic function. Notably, oral administration of fingolimod has been reported to alter synaptic function in the brain tissues of experimental autoimmune encephalomyelitis (EAE) mice (Gillingwater, 2012; Rossi et al., 2012).

In addition, fingolimod has been shown to reduce TNF-induced expression of B-cell activating factor (BAFF) and chemokine interferon-γ inducible protein 10 (CXCL10), which have immunoregulatory effects, in astrocytes (Hoffmann et al., 2015). This study revealed that the expression of CXCL10 was higher in the frontal cortices of AD patients than normal controls, suggesting that the abnormal increase in CXCL10 expression in AD is involved in the development and progression of the disease and that fingolimod could play a role in immune regulation in AD-affected brains.

In addition, this study found that one of the ways that fingolimod affects the brain tissue of AD patients could be through miRNAs. DUSP9, CCNT1, KSNJ10, LDLR, and FLT1 all have a large number of potential miRNA targets. This also suggests the importance of non-coding RNAs in the study of the pharmacological mechanism of fingolimod. More research on different types of non-coding RNAs is necessary. Moreover, neurons may contain an array of non-germline variants, including single-nucleotide variants (SNVs), copy number variants (CNVs), DNA breaks, inversions, and translocations. The methodology does not take into account the effects of these variables in a highly complex pathological context. This is one of the limitations of this study, and further research needs to be conducted from more perspectives to explore the function of fingolimod in the brain tissue of AD patients.

In this study, each of the two parts of Severity-related F-ADG-miRNAs and Severity-related F-ADGs was divided into two parts: the all sample group (MMSE0-27) and the mild or moderate dementia subgroup (MMSE 11-22). We found that fewer severity-related F-ADGs were found in the all sample group, GNAQ and MMP14, while more severity-related genes appeared in the mild or moderate dementia subgroup. We believe that two reasons may have contributed to this phenomenon. On the one hand, patients with excessive dementia may have interference from other complications, such as infections and metabolic diseases, which may have more effects on nerve cell gene expression levels beyond the pathological process of AD. On the other hand, the correlation between the expression level of F-ADGs and AD severity may only occur in some specific disease periods, rather than the entire course of AD. For example, some genes may only be positively correlated with disease severity in the early stage of AD, but not correlated or even negative correlated with the disease severity in the middle or late stages. Therefore, it is necessary to have the stratified study of AD patients at different stages of the disease on the basis of a comprehensive study of all patients with MMSE scores. A variety of MMSE scoring standards are used to grade the severity of dementia patients. An accepted way is: Normal cognition (24 and higher), Mild dementia (19–23), Moderate dementia (10–18), and Severe dementia (9 and lower) (Crum et al., 1993). Based on this classification method, our previous analysis of patients with 11–22 points can be regarded as a stratified analysis after the overall analysis, that is, a stratified analysis for mild dementia and moderate dementia. At the same time, we also tried to conduct separate stratified analysis on the “Severe dementia group” and the “no dementia group.” But in “Severe dementia” there are only two patients with 0 points and one patient with 3 points, and “no dementia” has only two samples of 26 points and 27 points. Neither can perform an ideal correlation analysis. Therefore, in this study, only the all sample group (MMSE 0-27) and the mild or moderate dementia subgroup (MMSE 11-22) were included.

### Limitations

This study has a number of limitations. First, this study is a bioinformatics analysis, and the predicted fingolimod drug targets are only potential drug targets. However, the *in vivo* effects of fingolimod on these predicted targets and the true impact on the pathogenesis of AD after treatment still need to be verified in further animal and clinical trials. Since the samples included in this study come from multiple databases, some clinical data, and MMSE scores may have been generated by different standards, which may cause bias and affect the results of the study. Moreover, neurons may contain an array of non-germline variants, including SNVs, CNVs, DNA breaks, inversions, and translocations. Therefore, in the future, additional large-scale and clinical studies of AD patients receiving fingolimod treatment are necessary.

## Conclusion

Fingolimod may affect the brain frontal cortex function of AD patients in many different ways, such as affecting immune cell infiltration, nerve cell or astrocyte proliferation, and GABAergic synapse function. miRNAs may also be involved. ALDH1L1, DUSP1, S1PR1, and GABBR2 may be core drug targets.

## Data Availability Statement

The original contributions presented in the study are included in the article/Supplementary Material, further inquiries can be directed to the corresponding author/s.

## Ethics Statement

Ethical review and approval was not required for the study on human participants in accordance with the local legislation and institutional requirements. Written informed consent for participation was not required for this study in accordance with the national legislation and the institutional requirements. Written informed consent was obtained from the individual(s) for the publication of any potentially identifiable images or data included in this article.

## Author Contributions

The study was conceived and designed by PY, YX, TW, DZ and GL. Data collection was carried out by PY. Processing and analysis of the imaging data was performed by PY and YX. PY led the statistical analysis. All authors contributed to interpretation of the data, preparation of the manuscript, and read and approved the final manuscript.

## Conflict of Interest

The authors declare that the research was conducted in the absence of any commercial or financial relationships that could be construed as a potential conflict of interest.
