# Supplementary figures and images for: The Therapeutic Targets of Fingolimod (FTY720) Are Involved in Pathological Processes in the Frontal Cortex of Alzheimer's Disease Patients: A Network Pharmacology Study
Source: Front Aging Neurosci. 2021 Feb 2;13:609679. doi: 10.3389/fnagi.2021.609679 (PMC7884771; doi:10.3389/fnagi.2021.609679)

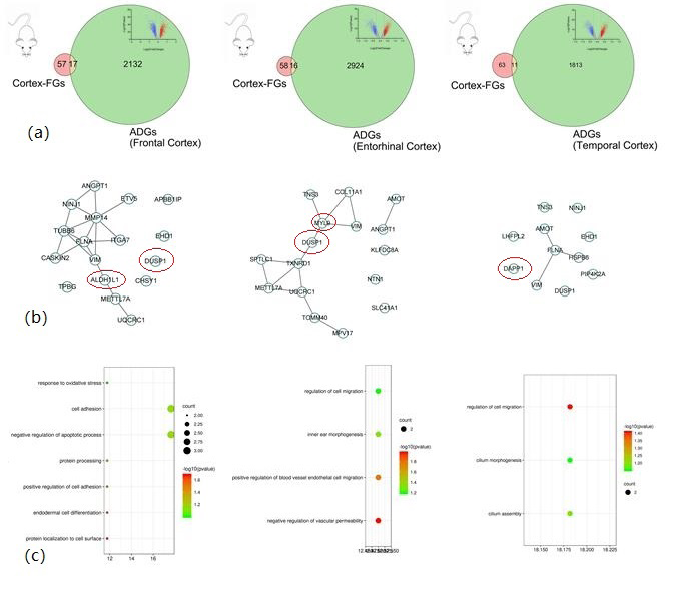

Supplement: Supplementary file 11 [file Image_2.TIF]
